# Impact of socioeconomic deprivation on screening for cardiovascular disease risk in a primary prevention population: a cross-sectional study

**DOI:** 10.1136/bmjopen-2015-009984

**Published:** 2016-03-21

**Authors:** Sarah-Jane Lang, Gary A Abel, Jonathan Mant, Ricky Mullis

**Affiliations:** 1 General Practice & Primary Care Research Unit, University of Cambridge, Cambridge, UK; 2 Cambridge Centre for Health Services Research, University of Cambridge, Cambridge, UK

**Keywords:** PRIMARY CARE, PREVENTIVE MEDICINE, PUBLIC HEALTH

## Abstract

**Objectives:**

Investigate the association between socioeconomic deprivation and completeness of cardiovascular disease (CVD) risk factor recording in primary care, uptake of screening in people with incomplete risk factor recording and with actual CVD risk within the screened subgroup.

**Design:**

Cross-sectional study.

**Setting:**

Nine UK general practices.

**Participants:**

7987 people aged 50–74 years with no CVD diagnosis.

**Methods:**

CVD risk was estimated using the Framingham equation from data extracted from primary care electronic health records. Where there was insufficient information to calculate risk, patients were invited to attend a screening assessment.

**Analysis:**

Proportion of patients for whom clinical data were sufficiently complete to enable CVD risk to be calculated; proportion of patients invited to screening who attended; proportion of patients who attended screening whose 10-year risk of a cardiovascular event was high (>20%). For each outcome, a set of logistic regression models were run. Crude and adjusted ORs were estimated for person-level deprivation, age, gender and smoking status. We included practice-level deprivation as a continuous variable and practice as a random effect to account for clustering.

**Results:**

People who had lower Indices of Multiple Deprivation (IMD) scores (less deprived) had significantly worse routine CVD risk factor recording (adjusted OR 0.97 (0.95 to 1.00) per IMD decile; p=0.042). Screening attendance was poorer in those with more deprivation (adjusted OR 0.89 (0.86 to 0.91) per IMD decile; p<0.001). Among those who attended screening, the most deprived were more likely to have CVD risk >20% (OR 1.09 (1.03 to 1.15) per IMD decile; p=0.004).

**Conclusions:**

Our data suggest that those who had the most to gain from screening were least likely to attend, potentially exacerbating existing health inequalities. Future research should focus on tailoring the delivery of CVD screening to ensure engagement of socioeconomically deprived groups.

**Strengths and limitations of this study**

- The study population represented all national Indices of Multiple Deprivation (IMD) deciles.
- Screening was administered and performed by research staff, reducing potential bias.
- The Framingham equation can underestimate cardiovascular disease (CVD) risk in the most deprived groups.
- Ethnicity could not be taken into account owing to incomplete recording in the medical records.

## Background

Cardiovascular disease (CVD) remains the leading cause of mortality worldwide^1^ and the second greatest cause of death in the UK.^2^ CVD is a major contributor to health inequalities, with a greater prevalence of CVD risk factors, incidence of events, poorer outcomes and premature death in people in lower socioeconomic groups. Despite an overall decline in CVD mortality, socioeconomic inequalities persist.^3^ Between 2010 and 2012, those living in the most deprived local authority^4^ were three times more likely to die prematurely of CVD compared with the most affluent.^2^ The cause of this health inequality is multifaceted and not completely explained by the higher burden of classical CVD risk factors.^5^ Cultural influences leading to poorer access to healthcare and suboptimal primary prevention management could contribute to the higher incidence of CVD in deprived groups. Currently, little evidence exists on the association between sociodemographic factors and routine CVD risk factor recording in primary care in the primary prevention population.

Modifiable risk factors for developing CVD such as elevated blood pressure (BP), serum cholesterol concentration and smoking have been shown to respond to pharmacological and behavioural interventions in primary^6^ ^7^ and secondary prevention of CVD. In 2009 in England, the National Health Service (NHS) Health Check programme was introduced for all people aged 40–74 years with no history of CVD or diabetes, with the aim of identifying those with previously unrecognised CVD risk and reducing health inequalities.^8^ ^9^ However, the impact of such a programme is dependent on uptake by those invited to attend and on the extent to which preventive treatments are applied in people identified as being at risk. Evidence from cervical,^10^ breast,^11^ colorectal^12^ and diabetic retinopathy^13^ screening demonstrates that socioeconomic deprivation is associated with lower levels of participation. Ensuring equitable uptake, or even targeting particularly those who are currently disadvantaged in terms of healthcare, is essential if these health inequalities are to be reduced, as inequitable attendance at screening has the potential to widen existing inequalities.^14^

This study examines the findings from a research-led programme of screening for CVD risk introduced across one socioeconomically diverse region of the UK. The aim of this study was to investigate whether socioeconomic deprivation is associated with completeness of routine CVD risk factor recording in primary care, with uptake of screening in people with incomplete risk factor recording and with actual CVD risk within the screened subgroup.

## Methods

### Data collection

Anonymised data were extracted from the primary care electronic health records of all patients aged between 50 and 74 years registered for over 12 months at nine general practices across the West Midlands area of the UK. Practices were purposefully selected to represent different practice sizes and levels of socioeconomic deprivation determined by the Indices of Multiple Deprivation (IMD) score of the practice area. All data queries were run between October 2008 and June 2009 using MIQUEST software. Extracted data included demographic information, cardiovascular risk factor details and records of prescribed medication (BP-lowering or cholesterol-lowering therapy). Patients who had pre-existing CVD recorded were excluded. The presence of cardiovascular risk factor details was defined as a non-zero value for BP and/or total or high-density lipoprotein (HDL) serum cholesterol in a value field alongside a read code for BP or total cholesterol recorded within the previous 5 years. We chose this time cut-off to reflect the recommended repeat frequency of the NHS Health Checks (every 5 years). All cholesterol-lowering and BP-lowering medication prescription data within the previous 90 days were extracted.

CVD risk was estimated using the Framingham equation, as recommended by the National Institute for Health and Care Excellence (NICE) at the time of data collection.^15^ This uses age, sex, BP, total/HDL cholesterol ratio, smoking status and existence of diabetes and/or left ventricular hypertrophy to estimate an individual's 10-year risk of having a cardiovascular event.^16^ CVD risk scores were adjusted by a factor of 1.4 or 1.5, respectively, for patients of South Asian origin or with a family history of premature cardiovascular events.^15^ Those with no recorded ethnicity were assumed not to be South Asian, and those with no recorded family history were assumed to have no family history of CVD. Those already receiving some form of preventive therapy were assumed to have been previously identified as high risk. Patients with diabetes were classified as high risk if they had at least one other recorded CVD risk factor.

Where there was insufficient clinical information to calculate CVD risk, patients were invited by letter to attend their practice for a screening assessment of their risk, with one reminder sent to non-responders 2 weeks later. A short covering letter that briefly summarised the study in a variety of languages and encouraged patients to speak to someone who can help them understand the study information was included with the invitation.

The screening assessment was carried out between January 2009 and May 2010 by research nurses at the patient's surgery. This included assessment of:

- Systolic and diastolic BP measurements
- Serum cholesterol (including total, HDL and low-density lipoprotein (LDL)), glucose and creatinine
- Medical history, including questions relating to CVD risk factors, existing CVD disease, family history of CVD and ethnicity
- Height and weight and calculated body mass index
- Ten-year CVD risk using the Framingham equation

Where patients were identified as being at high cardiovascular risk (ie, over 20% 10-year risk), the practice was informed. Further treatment was at the discretion of the general practitioner (GP).

### Statistical analyses

Three outcomes were explored. First, the proportion of patients for whom CVD risk factor recording was sufficiently complete to enable CVD risk to be calculated (without interpolation for missing data). Second, the proportion of patients invited to screening who attended. Third, the proportion of patients (restricted to those who attended a screening clinic) whose 10-year risk of having a cardiovascular event was high (above 20%) or low (20% or below).

For each outcome, a set of logistic regression models were run. In each case, the exposure of interest was person-level deprivation as defined by 2010 IMD deciles which were derived from whole country data and then applied to our dataset. In order to maximise power, this exposure was treated as a continuous variable taking integer values between 1 (least deprived national IMD decile) and 10 (most deprived national IMD decile). By using IMD decile as a continuous variable (rather than the underlying IMD score), we respect the rank-based interpretation of IMD while increasing power by assuming a linear relationship. For each model, departure for linearity was tested using a quadratic term; however, this was not significant in any models, so we present the simple linear versions here. As a sensitivity analysis, we also estimated models using a categorical version of patients' IMD which relaxed the linear relationship assumption. First, crude ORs were estimated from a logistic regression model with only a single exposure variable. This was done for deprivation as well as age group, gender and smoking status. As our prime interest was in deprivation, we also included a continuous variable for practice-level deprivation^17^ operationalised in the same way as individual deprivation and describing the deprivation of all patients attending the practice, rather than that of the patient. Following the crude analysis, adjusted ORs were estimated from a mixed-effects logistic regression including all variables described above. Practice was further included as a random effect (intercept) to account for clustering of individuals within the same practice. Analyses were restricted to patients with complete data for all covariates. Using the adjusted models, we estimate an OR comparing patients from the most deprived deciles with those from the least deprived deciles. As there are nine intervals between the most and least deprived deciles, the OR comparing them is equal to the OR for a single decile change raised to the power of nine. As a comparison, we also use the variance of the random effect to estimate an OR comparing those attending practices at the top and bottom (or mid) of the 95% reference range of practices. Essentially, this compares the range of practice influence (ignoring extreme practices).

Ethnicity was poorly coded, with 57% missing data, and was therefore not included in the original modelling. To determine whether this variable would influence the model, we ran a sensitivity analysis including complete case ethnicity codes. This had no significant effect on the strength of associations found within the model.

Data were analysed using Stata V.13.1 (StataCorp, Texas, USA).

## Results

**Figure 1:**
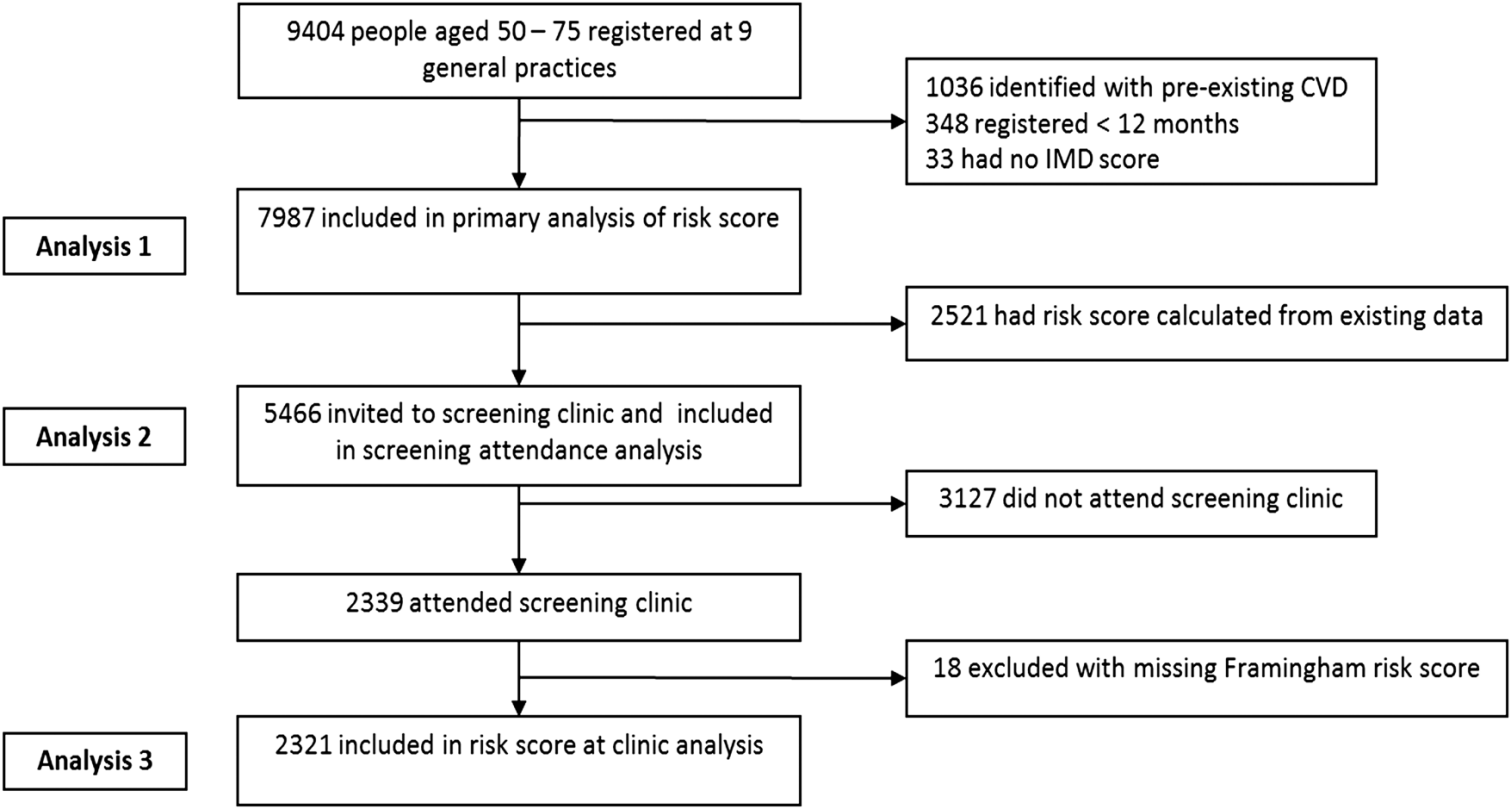
Flow chart depicting the number of patients identified from the nine general practices, the numbers included and the reasons for exclusion from each phase of the analyses.

Figure 1 depicts the number of patients identified from the nine general practices, the numbers included and the reasons for exclusion from each phase of the analyses. From the nine general practices involved, a total of 7987 people were included in the primary prevention analysis. Of these, 5466 (68%) had insufficient routinely collected clinical data to be able to calculate risk of CVD. All of these were invited to a screening clinic, of which 2321 (42%) attended, and 852 (37%) were subsequently found to be at high risk.

The mean age of the study population was 60 years, with approximately equal numbers of males and females (table 1). The distribution within each IMD decile was not uniform, with an excess of people in the most deprived decile and under-representation of people in some of the less deprived deciles.

**Table 1:** Demographic characteristics of the primary prevention population, aged 50–74 years

	IMD decile										
	1	2	3	4	5	6	7	8	9	10	Total
Primary prevention population (% of total)	940 (11.8)	272 (3.4)	226 (2.8)	615 (7.7)	659 (8.3)	1275 (16.0)	608 (7.6)	781 (9.8)	923 (11.6)	1688 (21.1)	7987 (100)
Mean age	60.5	60.4	60.4	59.8	60.3	59.9	60.3	59.2	60.2	59.9	60.2
Gender % male	46.7	45.6	48.7	44.9	48.4	49.6	46.1	48.9	50.2	49.6	48.4

IMD decile 1=least deprived, IMD decile 10=most deprived.

IMD, Indices of Multiple Deprivation.

**Figure 2:**
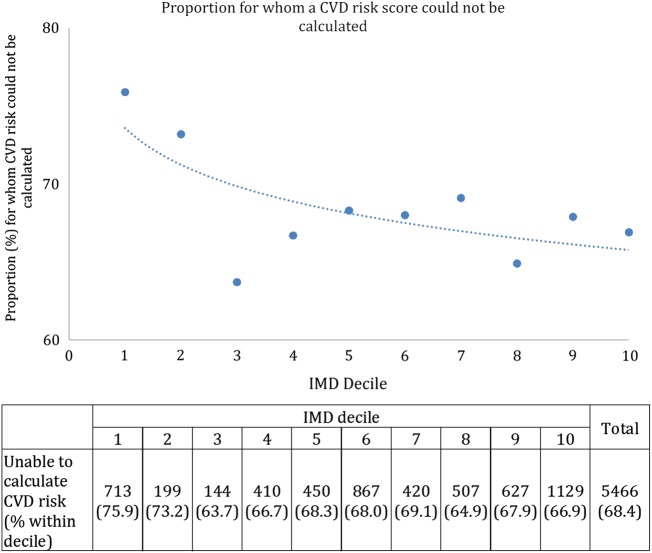
Proportion of the primary prevention population for whom a CVD risk score could not be calculated (without interpolation) owing to incomplete clinical data. (IMD decile 1=least deprived, IMD decile 10=most deprived.) CVD, cardiovascular disease; IMD, Indices of Multiple Deprivation.

A higher proportion of people in the two least deprived deciles had insufficient data in their records to allow a risk score to be calculated (figure 2).

**Figure 3:**
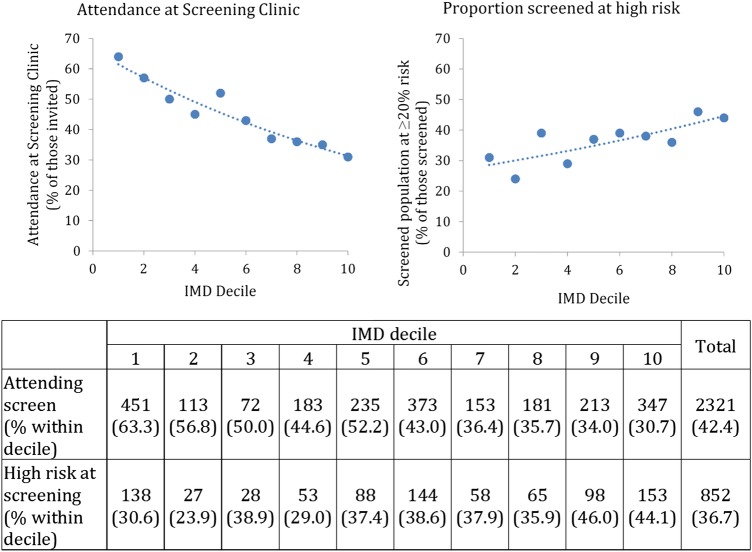
The percentage of the invited population who attended screening and the proportion of these found to be at high risk of CVD at the screening clinic. (IMD decile 1=least deprived, IMD decile 10=most deprived.) CVD, cardiovascular disease; IMD, Indices of Multiple Deprivation.

People with higher IMD scores (more deprived) were less likely to attend the screening clinic when invited (figure 3). However, of those who did attend, people with higher IMD scores were more likely to be at high risk of CVD.

Older people, non-smokers and those with higher IMD scores (more deprived) had significantly better CVD risk factor data recording in general practice (table 2).

**Table 2:** OR of association of IMD and other variables on having insufficient data to calculate a CVD risk score

	Not able to calculate CVD risk due to incomplete data			
(n=7987)	Crude OR (95% CI)	p Value	Adjusted OR (95% CI)	p Value
IMD (per decile)	0.97 (0.95 to 0.98)	<0.001	0.97 (0.95 to 1.00)	0.042
Age				
50–54	Reference	<0.001	Reference	<0.001
55–59	0.80 (0.69 to 0.92)		0.81 (0.70 to 0.93)	
60–64	0.64 (0.56 to 0.74)		0.64 (0.55 to 0.74)	
65–69	0.43 (0.37 to 0.50)		0.42 (0.36 to 0.49)	
70–74	0.35 (0.30 to 0.41)		0.35 (0.29 to 0.41)	
Practice IMD (per decile)	0.97 (0.95 to 0.98)	<0.001	0.99 (0.92 to 1.07)	0.829
Sex				
Female	Reference	0.675	Reference	0.184
Male	0.98 (0.89 to 1.08)		0.94 (0.85 to 1.03)	
Smoking status				
Ex/non-smoker	Reference	<0.001	Reference	<0.001
Current smoker	1.34 (1.18 to 1.51)		1.35 (1.19 to 1.54)	
Practice (SD of random effect)			0.29 (0.18 to 0.49)	<0.001

CVD, cardiovascular disease; IMD, Indices of Multiple Deprivation.

Of those invited to screening, females, non-smokers and the less deprived were more likely to attend (table 3).

**Table 3:** OR of association of IMD and other variables on attendance at screening

	Attendance at screening			
(n=5466)	Crude OR (95% CI)	p Value	Adjusted OR (95% CI)	p Value
IMD (per decile)	0.87 (0.85 to 0.88)	<0.001	0.89 (0.86 to 0.91)	<0.001
Age				
50–54	Reference	0.023	Reference	0.113
55–59	1.21 (1.04 to 1.40)		1.20 (1.03 to 1.40)	
60–64	1.26 (1.09 to 1.46)		1.15 (0.99 to 1.35)	
65–69	1.19 (0.99 to 1.41)		1.10 (0.91 to 1.32)	
70–74	1.10 (0.90 to 1.35)		0.99 (0.80 to 1.23)	
Practice IMD (per decile)	0.90 (0.88 to 0.91)	<0.001	0.96 (0.88 to 1.05)	0.418
Sex				
Female	Reference	<0.001	Reference	<0.001
Male	0.70 (0.63 to 0.78)		0.75 (0.67 to 0.84)	
Smoking status				
Ex/non-smoker	Reference	<0.001	Reference	<0.001
Current smoker	0.40 (0.35 to 0.47)		0.48 (0.42 to 0.56)	
Practice (SD of random effect)			0.35 (0.20 to, 0.63)	<0.001

IMD, Indices of Multiple Deprivation.

Of those who attended the screening clinic, it was the most deprived who were found to be most likely to be at >20% 10-year risk of CVD after adjusting for age, gender and smoking status (which are included in the Framingham risk score calculation). Practice IMD was not associated with CVD risk at screening, but there was a significant random effect of practice (table 4).

**Table 4:** OR of association of IMD and other variables on being at high risk at screening

	Likely to be found at high risk of CVD at screening clinic (n=2321)			
	Crude OR (95% CI)	p Value	Adjusted OR (95% CI)	p Value
IMD (per decile)	1.08 (1.05 to 1.11)	<0.001	1.09 (1.03 to 1.15)	0.004
Age				
50–54	Reference	<0.001	Reference	<0.001
55–59	1.89 (1.44 to 2.49)		2.73 (1.99 to 3.73)	
60–64	3.44 (2.64 to 4.47)		6.58 (4.80 to 9.04)	
65–69	6.73 (4.97 to 9.11)		14.10 (9.80 to 20.27)	
70–74	12.22 (8.49 to 17.58)		32.50 (21.19 to 49.85)	
Practice IMD (per decile)	1.06 (1.03 to 1.09)	<0.001	0.97 (0.91 to 1.04)	0.446
Sex				
Female	Reference	<0.001	Reference	<0.001
Male	4.44 (3.71 to 5.31)		6.79 (5.44 to 8.46)	
Smoking status				
Ex/non-smoker	Reference	<0.001	Reference	<0.001
Current smoker	3.54 (2.75 to 4.55)		5.73 (4.17 to 7.87)	
Practice (SD of random effect)			0.19 (0.08 to 0.43)	<0.016

CVD, cardiovascular disease; IMD, Indices of Multiple Deprivation.

We also compared the most deprived deciles with the least deprived deciles of the cohort. In terms of patients who had insufficient data to calculate a CVD risk score, there is a relatively modest effect of deprivation but not one small enough to be ignored (most vs least deprived decile OR (95% CI) 0.78 (0.62 to 0.99); p=0.042). The effect of deprivation was most dramatic for attendance at screening (most vs least deprived decile OR (95% CI) 0.34 (0.26 to 0.44); p<0.001). A substantial effect of deprivation was also seen for being at >20% 10-year risk of CVD among those screened, although, as noted above, in the opposite direction (most vs least deprived decile OR (95% CI) 2.12 (1.28 to 3.53); p<0.001). These estimates are broadly consistent with those achieved using a categorical variable for IMD (see online supplementary material S1). For all three outcomes, the effect of practice was substantial. Our estimate of ORs comparing practices at the top and bottom of the 95% reference range was 3.16 (95% CI 1.99 to 6.88, p<0.001) for having insufficient data to calculate a CVD risk, 4.00 (95% CI 2.19 to 11.63, p<0.001) for attendance at screening and 2.09 (95% CI 1.38 to 5.45, p=0.017). Thus, in the cases of being unable to calculate risk or attending screening, the effect of which practice a patient attended was potentially larger than the effect of deprivation.

10.1136/bmjopen-2015-009984.supp1 Supplementary data

## Discussion

### Summary of findings

Just over two-thirds of people at potentially high risk of developing CVD are not being routinely profiled in primary care when the opportunity arises, and within this group, there are differences related to socioeconomic deprivation. We found risk factor recording was better in older people, non-smokers and in people who were more deprived. The latter two results are surprising, in that one would have anticipated smokers to be more likely to have had their cardiovascular risk assessed. The higher recording in people who were most deprived may in part reflect the higher incidence of existing CVD and diabetes in these people. When people with insufficient risk factor data in their records were invited for screening, the less deprived were more likely to attend screening. Females and non-smokers were also more likely to attend screening. Of those who did attend screening, it was the most deprived who were most likely to be at high CVD risk. Therefore, our data suggest that those who potentially had the most to gain from the screening intervention were the least likely to take up the offer. It is also of note that which practice a patient attended had a strong influence of whether risk factors were recorded, whether invitees attended screening and the subsequently estimated risk.

### Comparison with existing literature

The few existing studies available support our findings that CVD risk recording, without the presence of an organised screening programme, is better in those subgroups that are at inherently higher risk, that is, the older and more deprived.^18^ ^19^ However, this appears to be the first paper to demonstrate that being a smoker is associated with poorer routine CVD risk recording. Artac *et al*^18^ assessed CVD risk factor recording prior to the implementation of the NHS Health Checks in an ethnically diverse primary care trust (PCT) in west London and found risk factor recording to be better in the most deprived patient group and BP and cholesterol recording to be better in older patients. This is consistent with the findings presented here using national IMD deciles to account for deprivation, which are potentially more representative of the national spectrum of deprivation compared with the local IMD tertiles used by Artac *et al*. Artac *et al*^18^ found that risk factor recording was better in women, which was not evident here. In addition, Artac *et al*^18^ were better able to examine the effect of ethnicity (78% data complete) on CVD risk factor recording, whereas here (43% data complete), this was not something that could be examined with confidence.

The existing literature on determinants of attendance at CVD screening is inconclusive. The literature can be divided into those that evaluate local screening programmes often set up as part of a research study, such as in this paper, and those that evaluate national externally implemented screening programmes, such as the NHS Health Checks.

#### Comparison with local screening programmes

Attendance at CVD screening is variable between countries and within the UK, ranging from 18%^20^ to 92%.^21^ This could be due to variation in the conduct of the studies and the target population, making comparison between studies difficult. This study's attendance rate of 42% sits well within this range. Our findings of non-attendance at screening being associated with those at higher risk, namely the most deprived, males and smokers, are consistent with previous studies evaluating local screening programmes, such as the OXCHECK study.^22^ The association between non-attendance and higher deprivation has been shown consistently in other studies.^23^ ^24^ Only Lambert *et al*^25^ did not find this, but their cohort contained only males, taken from the most deprived area nationally at the time.

#### Comparison with national screening programmes

The association between non-attendance at screening and the most deprived was not found in studies examining determinants of NHS Health Check coverage. This was seen both at a local level in east London,^26^ Stoke-on-Trent^27^ and Ealing^28^ and nationally,^29^ ^30^ with one national study finding the opposite association of increased coverage in more deprived PCTs (coefficient=−0.51 (95% CI −1.88 to 0.00) in the least deprived PCTs compared with those with the most, p=0.035).^30^ These findings could be explained by a number of methodological differences, such as targeting invites to those likely to be at higher risk, by using routinely collected data to estimate risk.^26–28^ It is not reported whether a greater proportion of the most deprived was invited, so intentional selection bias could have influenced the findings. These studies assessing local uptake were performed in deprived settings and examined the effect of local rather than national deprivation groupings; hence, the effect across the entire scale of deprivation may not be evident. The studies assessing NHS Health Check uptake at a national level used PCT^30^ or practice-level^29^ deprivation rather than deprivation of individuals, which could result in a distortion of the effect of deprivation. They also assessed coverage of the eligible population rather than proportion of those invited. While this is a suitable measure of assessment from a public health perspective, it fails to capture whether the lack of association between deprivation and attending an NHS Health Check is due to selection bias. Indeed, work undertaken in Germany^31^ and Singapore^32^ ^33^ has found that screening attendance is better in the most affluent groups.

### Strengths and limitations

A major strength of this study was that the study population represented all national IMD deciles, allowing comparison to be made over the entire spectrum of socioeconomic status. In addition, the screening was administered and performed entirely by research staff, ensuring that invitation method, availability of screening appointments, setting and content of screening were consistent across the entire study population. This would reduce potential selection or observer bias when delivering the screening programme. However, it is interesting to note that despite these controls, screening uptake still varied markedly between practices, even after adjusting for other factors.

The Framingham equation does not account for deprivation and has been shown to underestimate CVD risk for patients in the most deprived groups.^34^ Therefore, the true effect of deprivation may also have been underestimated here. The effect of ethnicity could not be accounted for in the regression model owing to incomplete recording in the medical records. We ran a sensitivity analysis including complete case ethnicity codes and found this had no tangible effect on the strength of associations. The absence of ethnicity recording in medical records has been reported previously^19^ ^35^ and is therefore a concern in many such studies.

### Implications for practice and research

Current incentives in primary care focus on achieving optimal management of those already known to be at high risk of developing CVD. However, many people at potentially high risk are not being routinely profiled in primary care when the opportunity arises. Incentivising general practices to identify and treat more of this group may be one way of attaining better population-level management.

While this study found that people in lower socioeconomic groups were more likely to have their CVD risk factors recorded, we also found that attempts to increase this proportion might lead to an exacerbation of health inequalities, via the inverse care law, as screening is not reaching the people who need it most.^36^ ^37^ This should be considered when designing and implementing screening programmes such as the NHS Health Check, as they are unlikely to reduce current health inequalities without active steps to address this issue. Future research should focus on how the delivery of CVD screening can be tailored to ensure engagement of socioeconomically deprived groups and people known to have risk factors for CVD. Work with simulation models suggests that targeted screening of deprived communities and family members of patients with CVD would be more cost-effective than mass screening.^38^ However, this approach has not been tested in a real-world study. Opportunistic screening of deprived patients when they present to primary care for other reasons^39^ or screening in a community setting such as religious or community centres,^40^ pharmacies^41^ and using community-based, lay health trainer-led approaches^42^ may be of value. Different approaches to recruitment should be directly compared with each other, in particular their impact on recruitment of socioeconomically deprived groups. One such trial is already underway, which will compare the use of a standard NHS Health Check invitation letter to (1) a question–behaviour effect questionnaire prior to the standard NHS Health Check invitation letter and (2) a financial incentive to complete the questionnaire.^43^
