# Supplementary material for: Impact of socioeconomic deprivation on screening for cardiovascular disease risk in a primary prevention population: a cross-sectional study
Source: BMJ Open. 2016 Mar 21;6(3):e009984. doi: 10.1136/bmjopen-2015-009984 (PMC4809080; doi:10.1136/bmjopen-2015-009984)
Supplement: Supplementary data [file bmjopen-2015-009984supp.pdf]

Appendix 1

Comparison of the effect of IMD from a set of models using a continuous variable for IMD decile and a set of models using a categorical variable for IMD decile. All three outcomes are shown.

Outcome	IMD decile	OR (95% CI) predicted from model using IMD decile as continuous	OR (95% CI) predicted from model using IMD decile as categorical
Insufficient data to calculate a CVD risk score	1	Reference	Reference
	2	0.97 (0.95, 1.00)	0.89 (0.75, 1.07)
	3	0.95 (0.90, 1.00)	0.94 (0.77, 1.14)
	4	0.92 (0.85, 1.00)	0.76 (0.61, 0.94)
	5	0.90 (0.81, 1.00)	0.79 (0.66, 0.94)
	6	0.87 (0.76, 0.99)	0.86 (0.69, 1.07)
	7	0.85 (0.72, 0.99)	0.87 (0.68, 1.12)
	8	0.83 (0.69, 0.99)	0.89 (0.64, 1.23)
	9	0.80 (0.65, 0.99)	0.87 (0.62, 1.24)
	10	0.78 (0.62, 0.99)	0.80 (0.59, 1.09)
Attendance at screening	1	Reference	Reference
	2	0.89 (0.86, 0.91)	0.89 (0.71, 1.10)
	3	0.79 (0.74, 0.83)	0.79 (0.62, 1.01)
	4	0.70 (0.63, 0.76)	0.75 (0.58, 0.97)
	5	0.62 (0.55, 0.70)	0.61 (0.49, 0.75)
	6	0.55 (0.47, 0.64)	0.45 (0.35, 0.58)
	7	0.48 (0.40, 0.58)	0.64 (0.47, 0.86)
	8	0.43 (0.35, 0.53)	0.47 (0.32, 0.69)
	9	0.38 (0.30, 0.49)	0.40 (0.28, 0.59)
	10	0.34 (0.26, 0.44)	0.35 (0.25, 0.49)
High CVD risk	1	Reference	Reference
	2	1.09 (1.03, 1.15)	0.92 (0.60, 1.40)
	3	1.18 (1.06, 1.32)	1.17 (0.74, 1.86)
	4	1.29 (1.08, 1.52)	1.37 (0.83, 2.25)
	5	1.40 (1.11, 1.75)	1.21 (0.82, 1.79)
	6	1.52 (1.14, 2.02)	1.31 (0.83, 2.05)
	7	1.65 (1.18, 2.32)	1.59 (0.91, 2.77)
	8	1.80 (1.21, 2.67)	1.36 (0.68, 2.72)
	9	1.95 (1.24, 3.07)	4.09 (2.03, 8.25)
	10	2.12 (1.28, 3.53)	1.99 (1.09, 3.60)
